# Guiding SPPs with *PT*-symmetry

**DOI:** 10.1038/srep14981

**Published:** 2015-10-08

**Authors:** Fan Yang, Zhong Lei Mei

**Affiliations:** 1 School of Information Science and Engineering, Lanzhou University, Lanzhou 730000, China; 2 State Key Laboratory of Millimetre Waves, Southeast University, Nanjing 210096, China

## Abstract

The concept of parity-time (*PT*) symmetry in SPPs is proposed and confirmed for the first time in this work. By introducing periodic modulation of the effective refractive index in SPP system, the asymmetric propagation is theoretically predicted and numerically demonstrated. After validation of this concept, we apply it in two applications: *PT*-waveguide and *PT*-cloak. Both two applications further illustrate the wide applicability of this concept in SPP system.

Surface plasmon polaritons (SPPs) is a kind of surface wave that propagates along the interface between metal and dielectric^1^. In optical frequency, the relative permittivity of metal is negative, which confines the electromagnetic (EM) wave on the surface of metal. The mechanism behind this confinement is the coupling between light wave and electrons in the metal^2^. The SPPs can concentrate and channel light using subwavelength structures, and this property makes it widely applicable in photonic circuits^3^,^4^, manipulation of light-matter interaction^5^, boosting of non-linear phenomena^6^ and sensors^7^,^8^. In recent years, many schemes have been proposed to control the propagation of SPPs. In 2010, two groups independently proposed that transformation optics (TO) can be used in controlling this surface wave (SPPs) at will^9^,^10^. Later, A. Vakil and N. Engheta demonstrated that the tunable graphene is also a good candidate for controlling propagation of SPPs^11^.

Moreover, tremendous attention has been paid on the parity-time(*PT*)-symmetric system in recent years. This concept was first proposed in quantum mechanics, and it was shown that the non-Hermitian Hamiltonians have real and positive energy spectrum if the *PT*-symmetry is satisfied^12^. In quantum system, a necessary condition for *PT*-symmetry is 
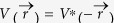
, where *V* is potential. Since the fundamental equations of EM wave and acoustic wave have the same form with Schrödinger equation, *PT*-symmetry has been also investigated in the field of optics and acoustics. For EM wave, the *PT*-symmetric system requires that the complex refractive index (optical potential) obeys the condition 
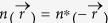
, which can be realized with balanced distribution of gain and loss media. *PT*-symmetric system enjoys many applications, like lossless propagation^13^,^14^,^15^,^16^, loss compensation^17^,^18^,^19^,^20^, unidirectional invisibility^21^,^22^, power oscillation^13^,^16^,^23^, etc. In nonlinear domain, *PT*-symmetry has been suggested to realize optical isolators and circulators^24^,^25^,^26^,^27^,^28^. For sound wave, the concept of *PT*-symmetric acoustics has been proposed recently^29^, and an acoustic sensor has been realized in the experiment using this scheme^30^.

In this letter, we propose the concept of *PT*-symmetry in SPPs. We show that asymmetric propagation of SPPs can be realized with *PT*-symmetric media. After demonstrating the validity of this concept, we apply it into two applications: *PT*-waveguide and *PT*-cloak. The excellent performance of asymmetric propagation in these applications further demonstrates that the concept of *PT*-symmetry will play an important role in SPP propagation.

## Results

### Principle

As we know, the metal behaves as an *ε*-negative media in optical frequency, and the interface between metal and air (or dielectric) supports propagation of SPPs. The frequency-dependent wave-vector of SPPs is^31^


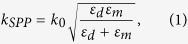


where *k*_0_ is the wave-vector in the free space, and *ε*_*d*_ and *ε*_*m*_ correspond to relative permittivity of dielectric and metal, respectively. The SPP wave can be considered as a wave propagating in a homogeneous medium with effective refractive index


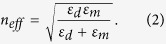


Then we will introduce a *PT*-symmetric modulation on the effective refractive index, which reads^21^


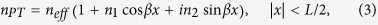


in which *n*_1_ represents the peak real index contrast, while *n*_2_ means the gain and loss periodic distribution. Generally, these amplitudes are much smaller than unity. *L* is length of this one-dimensional *PT*-symmetric system and *β* is modulation vector. The case for *n*_1_ = *n*_2_ corresponds to *PT*-symmetry breaking point. To achieve unidirectional invisibility at breaking point, *β* = 2*k*_*spp*_ is required, i.e. the modulation vector is twice the propagating wave-vector of SPPs.

By combining Equations (2) and (3), the required distribution of relative permittivity of SPP system can be sought in the form


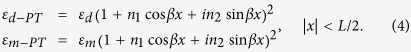


**Figure 1:**
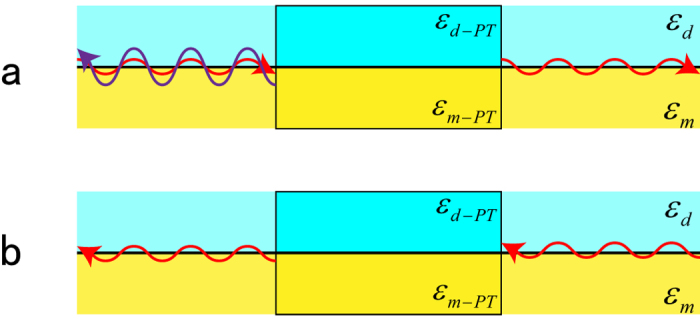
Schematic of *PT*-symmetry in SPPs. The light cyan and light yellow regions (upper and lower layers outside the box) are original dielectric and metal, and the dark cyan and dark yellow regions (upper and lower layers inside the box) are modulated ones. This periodic modulation will introduce nonreciprocal transmission of SPPs. The red curve corresponds to incident and transmitted SPP wave, and purple curve to reflected one. Asymmetric propagation for left and right incidence is presented.

This modulation of permittivity is adopted in the SPP system, as shown in Fig. 1, which will produce nonreciprocal transmission of SPPs along the interface between modulated metal and dielectric. In Fig. 1, the transmission property for unidirectional invisibility at breaking point is presented. For the incident SPP wave (red curve) coming from left, large Bragg reflection (purple curve) is observed in Fig. 1a. For the right incident one, shown in Fig. 1b, no reflection is observed and hence unidirectional invisibility is illustrated. This asymmetric property is attributed to the *PT*-symmetric modulation.

For unidirectional invisibility at breaking point, the reflection coefficients of SPP waves coming from left (*R*_*L*_) and right (*R*_*R*_) are different, they can be expressed as^21^


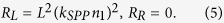


The transmission coefficient *T* is symmetric and equals unity in this condition. Therefore, this asymmetry of SPP system can be quantitatively controlled by adjusting the length *L*, modulation vector *β* and amplitude of modulation *n*_1_(*n*_2_).

### Numerical Simulation

**Figure 2:**
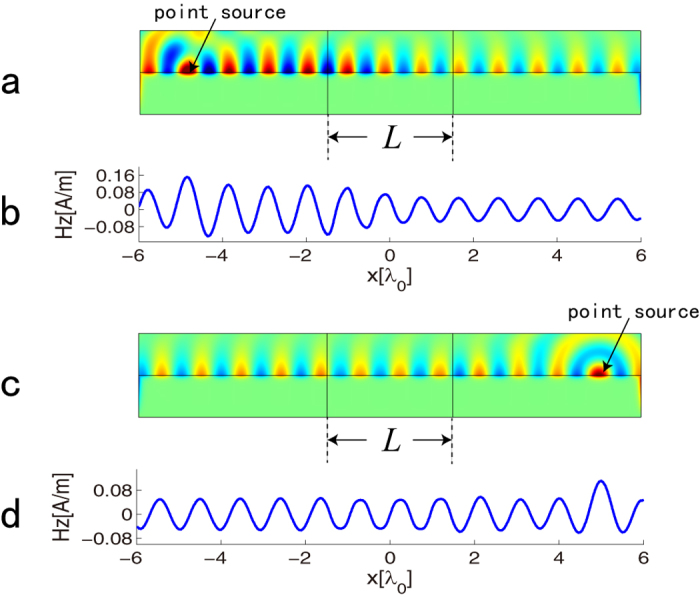
2D nonreciprocal transmission of SPPs. (**a**) Full wave simulation result for z-component of magnetic field *H*_*z*_ for left incident SPP wave; (**b**) numerical curve of *H*_*z*_ distribution along the interface of (**a**). (**c**) Full wave simulation result for z-component of magnetic field *H*_*z*_ for right incident SPP wave; (**d**) numerical curve of *H*_*z*_ distribution along the interface of (**c**).

As a demonstration of our concept, we consider the 2D nonreciprocal transmission of SPPs on the interface between dielectric (*ε*_*d*_ = 1, *μ*_*d*_ = 1) and metal (*ε*_*m*_ = −10, *μ*_*m*_ = 1), and these parameters will be used in all of following simulations. We denote free space wavelength as *λ*_0_, and the length of *PT*-modulation in Fig. 2 is *L* = 3*λ*_0_. The region of |*x*| < *L*/2 is the *PT*-symmetric media, where SPPs propagate along the interface between two modulated media with relative permittivity *ε*_*d*−*PT*_ and *ε*_*m*−*PT*_. In this simulation, *n*_1_ = *n*_2_ = 0.1 and modulation vector equals twice the wave-vector of SPPs, which means the system is working at the *PT*-symmetry breaking point. First, we set a source point at (−5*λ*_0_, 0) on the interface between dielectric and metal, and the simulation result of z-component of magnetic field *H*_*z*_ is shown in Fig. 2a. It is clear that there is large reflection (Bragg reflection) for the SPP wave coming from left. To give a numerical result, we extract the data of *H*_*z*_ along the interface, shown in Fig. 2b. It is obvious that the reflected SPP wave has overlapped with incident one. However, the reflection can hardly be observed for the right incident SPP wave when the source point is located at (5*λ*_0_, 0), shown in Fig. 2c. And the magnetic field *H*_*z*_ along the interface is shown in Fig. 2d, which demonstrates the unidirectional invisibility for SPP wave. In addition, the theoretical reflection coefficients are calculated using Equation (5), which are *R*_*L*_ = 3.95 and *R*_*R*_ = 0. Our simulated results show the corresponding two reflection coefficients are *R*_*L*_ ≈ 4 and *R*_*R*_ ≈ 0, respectively. Obviously, they are in accord with theoretical ones (See Methods for details).

**Figure 3:**
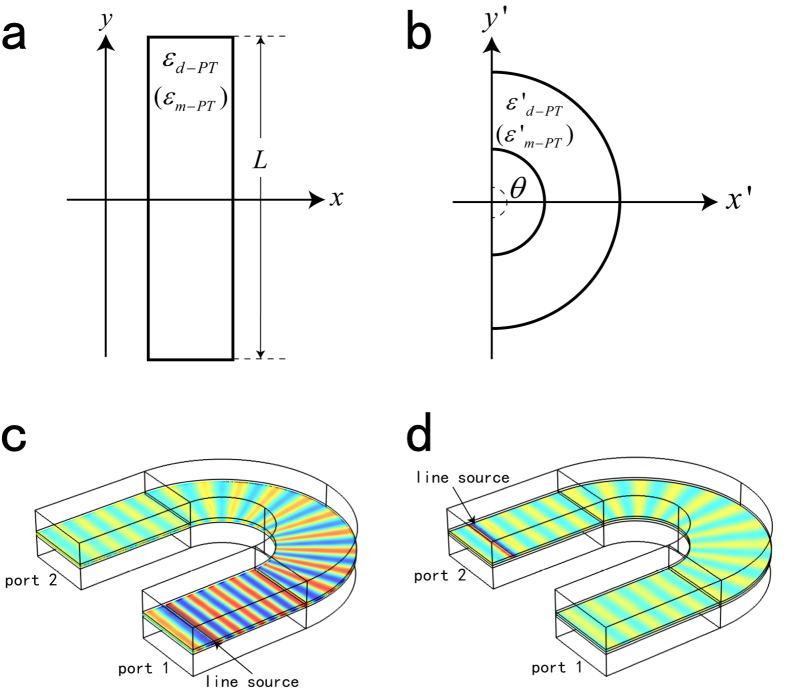
Schematic and simulation results of *PT*-waveguide for SPP wave. (**a**) A vertical strip like *PT*-symmetric system in virtual space; (**b**) a half ring like system in physical space. The parameters are marked out in (**a**,**b**). (**c**) Full wave simulation result for z-component of electric field *E*_*z*_ for SPP wave excited at port 1; (**d**) full wave simulation result for z-component of electric field *E*_*z*_ for SPP wave excited at port 2.

The above simulations have validated the concept of *PT*-symmetry in SPPs. Then we will consider two *PT*-symmetric devices for SPP wave, which is of great significance in application. The first application is a *PT*-waveguide for SPP wave, which can bend the SPP wave nonreciprocally. To achieve this goal, a transformation is required, which is expressed as *x*′ = *x*cos(*θy*/*L*), *y*′ = *x*sin(*θy*/*L*), *z*′ = *z*^32^. It maps a vertical strip like *PT*-symmetric system shown in virtual space (see Fig. 3a) into a half ring like system in physical space (see Fig. 3b). The EM parameters of half ring after transformation are written as^32^


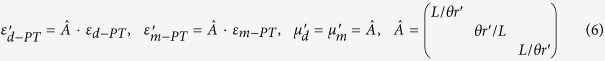


in which 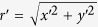 and 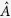 is expressed in cylindrical coordinate system (*r*, *ϕ*, *z*). The parameters are set as *L* = 10*λ*_0_, *θ* = *π* and *n*_1_ = *n*_2_ = 0.05 in the simulation. Since the interface between *ε*-negative and *ε*-positive media supports TM mode (the electric field is perpendicular to the interface), the simulation results of z-component of electric field *E*_*z*_ on the interface between dielectric and metal are considered and presented in Fig. 3c,d. In Fig. 3c, the SPP wave is excited using a magnetic line current at port 1, in which large reflection of SPP wave exists. However, there is merely no reflected SPP wave for the excitation at port 2 shown in Fig. 3d. Thus, a *PT*-waveguide for SPP wave is realized.

**Figure 4:**
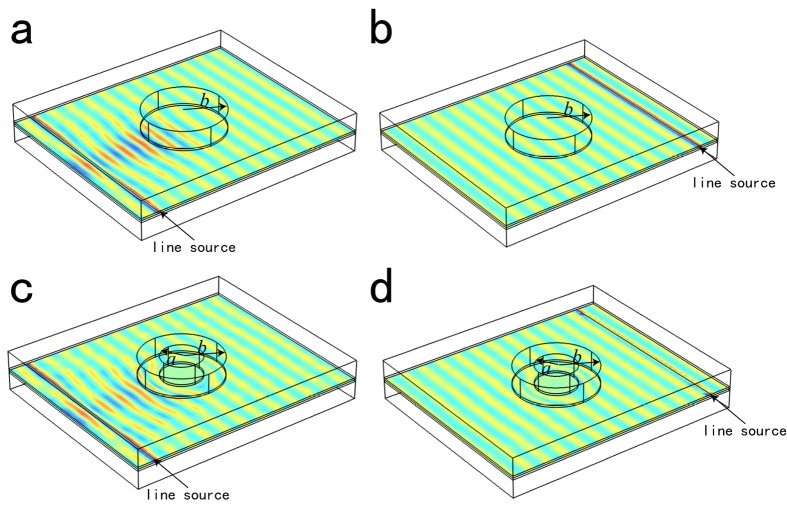
Full wave simulation results of *E*_*z*_ for *PT*-cloak. (**a**,**b**) when a periodic complex modulation is applied in the region *r* ≤ *b* in virtual space, (**a**,**b**) correspond to excitation at two sides. (**c**,**d**) The corresponding physical space of (**a**,**b**), in which the region *r* ≤ *a* is unidirectionally cloaked.

As a second application, we design a 3D cylindrical *PT*-cloak for SPP wave. In cylindrical coordinate system (*r*, *ϕ*, *z*) of virtual space, we apply a periodic complex modulation in the region *r* ≤ *b* (*b* = 2*λ*_0_) by Equation (4) with *n*_1_ = *n*_2_ = 0.1 and *β* = 2*k*_*spp*_. This modulation leads to the asymmetric propagation of SPP wave for left and right incidences, which are shown in Fig. 4a,b. In this simulation, the propagation of SPPs is in x-y plane.

Then a transformation is applied which can be expressed as *r*′ = *f*(*r*) = (*b* − *a*)*r*/*b* + *a* for *a* ≤ *r* ≤ *b* (*a* = *λ*_0_), *θ*′ = *θ* and *z*′ = *z*^33^. After this coordinate transformation, the tensorial EM parameters in physics space *a* ≤ *r* ≤ *b* is same as Equation (6), except^33^


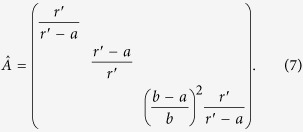


By setting the EM parameters as in Equation (7), we can achieve a *PT*-cloak for SPP wave. And the unidirectional invisibility for SPP wave in physical space is realized. The simulation results for left and right incident SPP wave are presented in Fig. 4c,d, respectively, in which large Bragg reflection for left incidence and invisibility for right incidence are visualized. Similar nonreciprocal propagation of SPP wave is revealed in this device, which further demonstrates the correctness of our scheme. It also should be mentioned that this *PT*-cloak for SPP is a one-way cloak, i.e., the cloaked object can be detected for the left incident SPP wave, but cannot for the right incident one.

## Discussion

**Figure 5:**
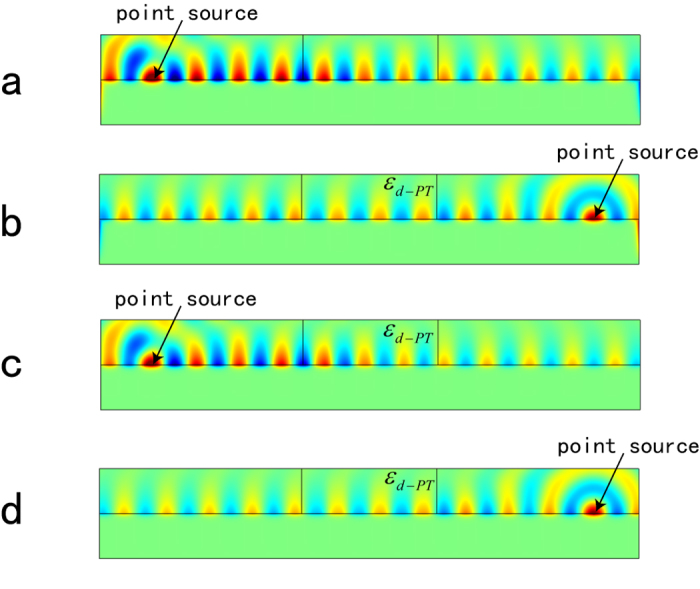
Full wave simulation results of simplified SPP system. (**a**) The distribution of *H*_*z*_ for left incident SPP wave; (**b**) The distribution of *H*_*z*_ for right incident SPP wave. (**c**,**d**) Simulation results when loss of metal is introduced in (**a**,**b**). Note that the metal part is not modulated in this simulation.

In above design and simulation, both dielectric and metal are periodically modulated. Indeed, this is not required and can be simplified in the real application. Since the skin depth of SPP wave in the metal is much smaller than that in dielectric, most EM field will distribute on the dielectric side. In this condition, the modulated metal plays a much smaller role in asymmetric propagation of SPPs. Thus the modulation of both dielectric and metal can be reduced to that of dielectric. To verify this simplification, some additional simulations have been done, as shown in Fig. 5a,b. These two simulations are exactly the same as these in Fig. 2a,c, except for this simplification. As can be seen in the figure, nearly same asymmetric properties are presented, though the metal is not modulated. Similarly, the modulation of metal can also be removed for the *PT*-waveguide and *PT*-cloak, even some other *PT* devices for SPP system. This simplification will make *PT*-symmetry in SPPs easier to implement in the real application.

In general, SPPs will attenuate in the propagation owning to the losses arising from absorption in the metal. Since loss of metal is much larger than dielectric, it plays a more important role in the propagation of SPPs. Taking these into account, we introduce a small loss in the metal (*ε*_*m*_ = −10 − 0.5*j*) for simplified model. The corresponding simulations are presented in Fig. 5c,d. Obviously, the loss does not affect the nonreciprocal transmission and unidirectional invisibility of SPPs. It demonstrates that relatively small losses will not deteriorate the performance of *PT*-symmetric system in practical applications.

The balanced distribution of gain in *PT*-symmetric system is difficult in terms of realization. A method to realize this system is to use Fe-doped LiNbO_3_^16^, Er^3+^-doped silica^27^, etc. Moreover, *PT*-symmetric property remains in the pseudo-Hermitian *PT* system with only absorptive media^34^,^35^, which makes the *PT*-symmetric system of SPPs much more realizable.

## Methods

### Simulation Settings

All the numerical simulation results are obtained using the finite element solver COMSOL Multiphysics. For the two dimensional simulation (Figs 2 and 5), the scattering boundary were set for four sides, and the point source is set as magnetic current on the interface between dielectric and metal. For three dimensional simulation (Figs 3 and 4), the curved outer boundary in Fig. 3 and the boundary perpendicular to line source in Fig. 4 are set as PMC, which makes the magnetic field perpendicular to these boundaries. The other boundaries are set as scattering boundary condition. The line source excitation is set as magnetic current locates on the interface between dielectric and metal, and is parallel to y-axis.

### Calculation of Reflection Coefficients

**Figure 6:**
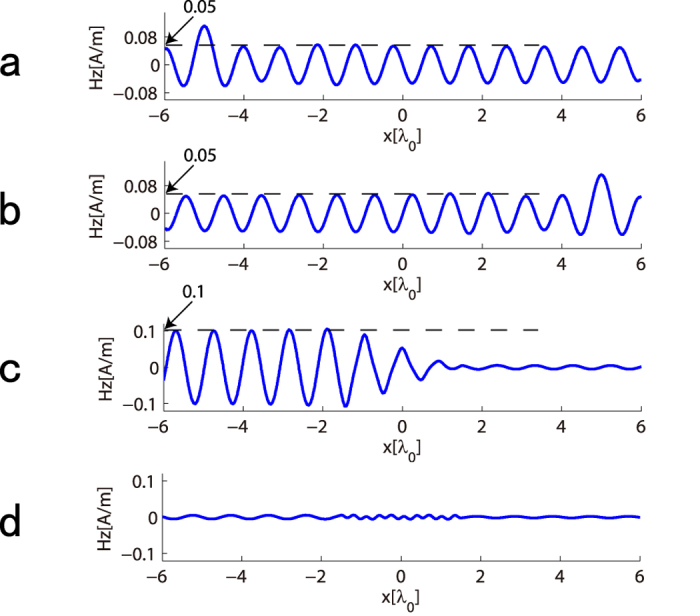
Calculation of reflection coefficients. (**a**,**b**) Numerical curve of *H*_*z*_ distribution along the interface for no modulation (incident SPP wave distribution) for left (**a**) and right (**b**) incidence. (**c**,**d**) Reflected SPP wave distribution for left (**c**) and right (**d**) incidence.

Since the excitation is a point source, the *S* parameters (*S11* and *S22*) can not be obtained directly from simulation of Fig. 2. In view of this, a simulation for no modulation of Fig. 2 is done, i.e. the incident wave. The magnetic fields *H*_*z*_ along the interface for left and right incidence are shown in Fig. 6a,b. Afterwards, the reflected wave, shown in Fig. 6c,d, can be obtained by subtracting unmodulated wave from modulated one. It is apparent that the amplitude of reflected wave is about twice that of incident one (*r*_*L*_ ≈ 2) for left incidence, while approximates 0 (*r*_*R*_ ≈ 0) for right incident one. So, the reflection coefficients are 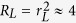 and 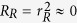.

## Additional Information

**How to cite this article**: Yang, F. and Lei Mei, Z. Guiding SPPs with *PT*-symmetry. *Sci. Rep*. **5**, 14981; doi: 10.1038/srep14981 (2015).
